# Nicotinic acetylcholine receptors modulate osteoclastogenesis

**DOI:** 10.1186/s13075-016-0961-x

**Published:** 2016-03-12

**Authors:** Peter Mandl, Silvia Hayer, Thomas Karonitsch, Petra Scholze, David Győri, Despoina Sykoutri, Stephan Blüml, Attila Mócsai, Gyula Poór, Sigismund Huck, Josef S. Smolen, Kurt Redlich

**Affiliations:** Division of Rheumatology, Medical University of Vienna, Vienna, Austria; CeMM Research Center for Molecular Medicine of the Austrian Academy of Sciences, Vienna, Austria; Center for Brain Research, Medical University of Vienna, Vienna, Austria; Department of Physiology, Semmelweis University, Budapest, Hungary; National Institute of Rheumatology and Physiotherapy, Budapest, Hungary

**Keywords:** Osteoclastogenesis, Acetylcholine receptor, Nicotine

## Abstract

**Background:**

Our aim was to investigate the role of nicotinic acetylcholine receptors (nAChRs) in in-vitro osteoclastogenesis and in in-vivo bone homeostasis.

**Methods:**

The presence of nAChR subunits as well as the in-vitro effects of nAChR agonists were investigated by ex vivo osteoclastogenesis assays, real-time polymerase chain reaction, Western blot and flow cytometry in murine bone marrow-derived macrophages differentiated in the presence of recombinant receptor activator of nuclear factor kappa B ligand (RANKL) and macrophage colony-stimulating factor (M-CSF). The bone phenotype of mice lacking various nAChR subunits was investigated by peripheral quantitative computed tomography and histomorphometric analysis. Oscillations in the intracellular calcium concentration were detected by measuring the Fura-2 fluorescence intensity.

**Results:**

We could demonstrate the presence of several nAChR subunits in bone marrow-derived macrophages stimulated with RANKL and M-CSF, and showed that they are capable of producing acetylcholine. nAChR ligands reduced the number of osteoclasts as well as the number of tartrate-resistant acidic phosphatase-positive mononuclear cells in a dose-dependent manner. In vitro RANKL-mediated osteoclastogenesis was reduced in mice lacking α7 homomeric nAChR or β2-containing heteromeric nAChRs, while bone histomorphometry revealed increased bone volume as well as impaired osteoclastogenesis in male mice lacking the α7 nAChR. nAChR ligands inhibited RANKL-induced calcium oscillation, a well-established phenomenon of osteoclastogenesis. This inhibitory effect on Ca^2+^ oscillation subsequently led to the inhibition of RANKL-induced NFATc1 and c-fos expression after long-term treatment with nicotine.

**Conclusions:**

We have shown that the activity of nAChRs conveys a marked effect on osteoclastogenesis in mice. Agonists of these receptors inhibited calcium oscillations in osteoclasts and blocked the RANKL-induced activation of c-fos and NFATc1. RANKL-mediated in-vitro osteoclastogenesis was reduced in α7 knockout mice, which was paralleled by increased tibial bone volume in male mice in vivo.

**Electronic supplementary material:**

The online version of this article (doi:10.1186/s13075-016-0961-x) contains supplementary material, which is available to authorized users.

## Background

Acetylcholine (ACh) was the first neurotransmitter to be discovered [1]. It is an essential mediator of both the central and the peripheral nervous system [2, 3]. ACh acts on two main types of receptors: nicotinic acetylcholine receptors (nAChRs), which are ligand-gated ionotropic receptors, and muscarinic acetylcholine receptors (mAChRs), which are metabotropic G-protein coupled receptors [4]. Neuronal nAChRs are pentamers composed of either homomeric or heteromeric combinations of twelve different nicotinic receptor subunits: α2 through α10 and β2 through β4 [5]. In addition to its role in the nervous system, ACh is increasingly recognized as an important modulator in various non-neuronal tissues [6–8].

nAChRs have also been identified on cells known to be essential for the maintenance of bone homeostasis, such as osteoblasts and osteoclasts [9–12]. In addition, other components involved in cholinergic transmission, including the vesicular ACh transporter, but also the enzyme choline acetyltransferase needed for the synthesis of Ach, have been reported to be present in murine osteoblasts [13]. Moreover, recent studies have revealed the presence of cholinergic innervation in bone marrow, suggesting a potential regulatory role of ACh on all bone marrow cells including osteoclasts, but possibly also osteoblasts [12, 14, 15].

Osteoclasts are formed by the fusion of hematopoetic mononuclear cells [16]. This process requires the presence of macrophage colony-stimulating factor (M-SCSF) and receptor activator of nuclear factor kappa B ligand (RANKL) [16]. Recently, several studies have implicated nAChRs and mAChRs in regulating osteoclastogenesis; however, their effects on osteoclastogenesis have not yet been fully elucidated [17–20]. nAChR agonists, such as nicotine or carbamylcholine, have been shown to reduce the formation of osteoclasts in vitro [12, 17], while other studies have reported a stimulating effect of nAChR agonists on osteoclastogenesis [18, 21, 22]. Moreover, nicotine was also shown to affect osteoblast differentiation and function [23–25]. Mice lacking the α2 nAChR subunit or the M2 mAChR have increased bone resorption and low bone mass due to increased osteoclastogenesis [12, 19], while female α7 knockout mice exhibited significantly increased bending stiffness and cortical thickness, as well as reduced gene expression of the osteoclast marker cathepsin K [20].

The potential relevance of cholinergic regulation in bone homeostasis under pathological conditions, such as chronic inflammation, was further highlighted by investigations into α7-deficient mice which exhibit increased bone loss as compared to wild-type (WT) mice in an animal model of arthritis [26].

Our aim was to investigate the role of nAChRs in osteoclasts in detail, and to examine the effect of these receptors on osteoclastogenesis and bone turnover in WT and knockout mice lacking individual nAChR subunits.

## Methods

### Mice

Experiments were performed on C57Bl/6 (WT) mice and mice with deletions of the nAChR subunit genes α7 [27], β2 [28] and β4 [29]. Mice used in this study were backcrossed onto C57Bl/6 background for 6 (β4), 7 (α7) or 12 (β2) generations after germ line transmission and kept at the Center for Brain Research, Medical University of Vienna, Austria. All data were generated from sex- and age-matched littermates. The Institutional Animal Care and Use Committee of the Medical University Vienna approved all mouse procedures.

### Osteoclastogenesis from bone marrow-derived macrophages

Bone marrow cells obtained from the femurs and tibias of wild-type and knockout mice were cultured in alphaMEM medium (Gibco) containing 10 % fetal calf serum and 1 % penicillin-streptomycin. For in vitro osteoclast formation, we used mouse bone marrow cells stimulated in Petri dishes for 3 days with M-CSF 10 ng/ml (bone marrow macrophages; BMMs). BMMs were subsequently plated on 96-well plates and supplemented with 30 ng/ml M-CSF and 50 ng/ml RANKL with media/cytokine changes every 2 days. After 5–6 days under these osteoclastogenic conditions, we performed staining for tartrate-resistant acidic phosphatase (TRAP) and imaged the plates using an inverted microscope. The number of osteoclasts (OCs), defined as TRAP-positive cells containing ≥3 nuclei (TRAP-positive multinucleated cells), was counted manually in each well, while the number of cells containing <3 nuclei (TRAP-positive or TRAP-negative mononucleated cells) were counted in three power fields in each well and expressed as percent of control from at least three independent cultures. All agonists and antagonists were administered to BMMs together with M-CSF and RANKL unless otherwise noted.

### In vivo osteoclastogenesis

Calvarial bone resorption was induced by subcutaneous injection of lipopolysaccharide (LPS; Sigma) above the calvaria into 7-week-old C57Bl/6 mice. The heads of anesthetized mice were shaved to receive subcutaneous injections above the calvaria in the midline of the skull located between the ears and eyes. A total of 20 mice were divided into four groups: group 1 (*n* = 5), injections of 500 μg/ml LPS and on alternate days an equivalent dose of phosphate-buffered saline (PBS); group 2 (*n* = 5), injections of 500 μg/ml LPS together with 4 mg/kg nicotine (in a single volume of 100 μl) and on alternate days 4 mg/kg nicotine alone (100 μl); group 3 (*n* = 5), daily injections of 4 mg/kg nicotine (100 μl); and group 4 (*n* = 5) daily injections of PBS (100 μl) for a period of 1 week. Animals were sacrificed by cervical dislocation; calvariae were removed and suspended in phosphate-buffered formalin for 6 hours. Following 7 days of decalcification in ethylenediaminetetraacetic acid (EDTA), tissues were embedded in paraffin, and serial sections were stained for TRAP (Sigma). Osteoclast numbers and eroded areas were determined as previously described [30] by Osteomeasure**®** software.

### Immunocytochemical staining

BMMs stimulated with M-CSF and RANKL were cultured on 12-mm plain glass coverslips and stained with anti-choline acetyl transferase antibody (AB144P; Millipore, 1:25) for 1 hour, followed by a secondary, biotinylated rabbit-antigoat IgG (Vector) for 30 minutes, avidin/biotin complex (Vector) for 45 minutes and diaminobenzidine substrate (Vector) for 10 minutes. Coverslips were finally stained with Mayer’s hematoxylin solution and were mounted on glass slides.

### Quantitative and semi-quantitative real-time polymerase chain reaction

For investigating the presence/absence of nAChR subunits we performed semi-quantitative real-time polymerase chain reaction (RT-PCR) analysis while quantitative RT-PCR analysis was undertaken to investigate changes in RANKL-dependent genes. Nucleus interpeduncularis was homogenized using an Ultra-Turrax-Disperser. Messenger RNA was extracted from BMMs stimulated with M-CSF and RANKL as well as nucleus interpeduncularis homogenate using the RNeasy Kit (Qiagen). RNA quality was ascertained by measuring absorption at 260 and 280 nm and calculating the A260/A280 ratio, which was 1.8–2.0 for the RNA samples used in our experiments. Care was taken when creating complementary DNA (cDNA) templates to obtain libraries free of inhibitors. Reaction efficiency for the housekeeping gene glyceraldehyde-3-phosphate dehydrogenase as well as all genes of interest was ensured by the creation of standard curves using measurements obtained over a dilution range. A volume of 800 nl/1 μl cDNA was used for the semi-quantitative/quantitative PCR, respectively. For the semiquantitative RT-PCR, cDNA was amplified using REDTaq-DNA Polymerase (Sigma-Aldrich) and the primers listed in Additional file 1: Table S1 were analyzed visually on an acrylamide gel. Quantitative RT-PCR expression of mRNA was detected and quantified using SYBR Green PCR Master Mix (Applied Biosystems). The following primers were investigated (*genes*): nicotinic receptor subunits: α2-7, 9, 10 as well as β2–4 (see Additional file 1: Table S1 for primer sequences), cathepsin K (*Ctsk*), matrix-metalloprotease 9 (*Mmp9*), tartrate-resistant acidic phosphatase (*Acp5*), nuclear factor of activated T cells c1 (*Nfatc1*), and receptor activator of nuclear factor kappa beta (*Tnfrsf11a*).

### Bone morphology

We performed histomorphometry using the Osteomeasure**®** software as previously described [30] in 16-week old male β4–/– and α7–/– mice as well as WT age-matched littermates. We measured bone mineral density (BMD) by peripheral quantitative computed tomography (pQCT) with an XCT Research M+ pQCT machine (Stratec Medizintechnik). We measured three slices in the proximal tibia and calculated BMD values as the mean of three slices, using a voxel size of 0.07 mm and a threshold of 400 mg/cm^3^.

### Flow cytometry and MTT assay

For flow cytometric analyses, we used a BD FACS Canto II (BD). We stained for CD11b (AM1/70), GR1 and F4/80 (all from BD Biosciences). We characterized osteoclast precursors as (CD11b^high^)/(GR1^low^) cells. For apoptosis evaluation, M-CSF- and RANKL-stimulated BMMs were stained for Annexin V (Axxora LLC) and 7-Aminoactinomycin-D (7-AAD) (Fluka Sigma-Aldrich) [31]. We characterized live cells as Annexin V low/7-AAD low, apoptotic cells as Annexin V high/7-AAD low, and dead cells as Annexin V high/7-AAD high, respectively. To distinguish from small cellular debris, forward and right angle scatter gates have been defined by contour plot and the probability scaling method, where regions were drawn to contain 95 % of the relevant lymphoid and myeloid cell populations. Gates were limited in their forward angle scatter dimension not to contain events falling in channel numbers higher than 250,000, thereby eliminating the majority of cellular clusters or multiplets. Acquisition was stopped when 10,000 events within a separate lymphoid region had been reached. The number of myeloid cells acquired was in proportional relations between equivalent cultures, albeit slightly varying by culture conditions. Percentages were read out as precentage of parent, where the parent region comprised of lymphoid and myeloid cell populations not containing small cellular debris or large cellular clusters. Thresholds for staining positivity were determined using unstained cell preparations by just omitting the addition of fluorescein-isothiocyanate (FITC)-conjugated Annexin-V or 7-AAD as a viability dye. For each antibody, appropriate isotype-matched control staining was performed to distinguish positive staining from non-specific noise. We used the following fluorophores: Annexin V: FITC; CD11b and F4/80: Allophycocyanine; and GR-1: R-phycoerythrine. Cytotoxicity was evaluated using a standard MTT assay (Merck Millipore) by measuring absorbance on an enzyme-linked immunosorbent assay (ELISA) plate reader.

### Calcium measurement

Mouse bone marrow cells were first cultured for 2 days in the presence of recombinant mouse M-CSF (10 ng/ml) (BMMs). Non-adherent cells were then plated at 2 × 10^5^ cells/cm^2^ density and cultured in the presence of 50 ng/ml recombinant mouse M-CSF and 50 ng/ml mouse RANKL for 72 hours. For Ca^2+^ measurements, the cells were incubated with 5 μM Fura-2-AM (Tocris Bioscience) and 0.05 % pluronic F127 (Sigma-Aldrich). Fluorescence intensity was measured using excitation wavelengths of 340 and 380 nm, and the emitted fluorescence at 510 nm (fluorescence ratio 340/380 nm). Changes in the fluorescence ratio 340/380 nm are shown as a function of time. Evaluation of the images was performed with MetaFluor**®** software (Molecular Devices). The images were scanned and plotted with an interval of 5 seconds. Graphs are representative of four independent cells from three independent experiments.

### Western blotting

BMMs stimulated with 30 ng/ml M-CSF and 50 ng/ml RANKL were lysed in radioimmunoprecipitation buffer (RIPA-buffer; 10 mM Tris-HCl, pH 7.5, 150 mM NaCl, 1 % NP-40, 1 % sodium deoxycholate, 0.1 % SDS, 1 mM EDTA) supplemented with protease inhibitors (Roche). Lysates were cleared by centrifugation in a microcentrifuge. Protein content was determined using the Bradford protein assay (Bio-Rad). To reduce and denature the protein, samples were boiled in Laemmli sample buffer. Lysates were resolved by SDS-PAGE. This was followed by electrotransfer onto nitrocellulose membrane, with a loading amount of 30 μg protein. After blocking with 5 % milk, membranes were incubated with primary antibodies NFATC1 (Santa-Cruz), c-FOS (Cell Signalling) and Actin (Cytoskeleton) according to the manufacturer’s protocol and then exposed to horseradish peroxidase-conjugated secondary antibodies (Jackson). Specific bands were detected with the ECL-detection kit (Pierce) on Amersham Hyperfilm ECL (GE Healthcare). Protein expression was quantified using Image J® software.

### Statistical analyses

All experiments were performed at least three times (or on at least three individual mice) with comparable results. Data are presented as bar graphs showing the mean ± standard deviation (SD). Statistical analysis was performed using one-way analysis of variance with Dunnett's post-hoc test for comparisons involving more than three groups and Student’s two-population unpaired *t*-test for comparisons involving two groups, both performed using GraphPad Prism**®** version 4.00 for Windows (GraphPad Software, San Diego California, USA). Asterisks in figures indicate the following *p*-values: *p < 0.05; **p < 0.005; ***p < 0.001.

## Results

### Osteoclast formation is inhibited by nAChR ligands in vitro

**Fig. 1:**
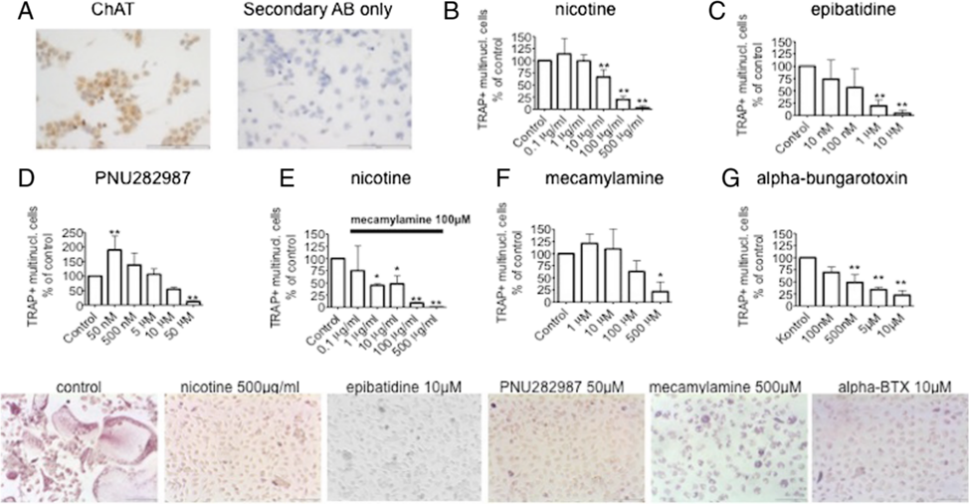
Osteoclast formation is inhibited by nAChR ligands in vitro. **a** (*Left panel*) Immunocytochemical staining of choline acetyl transferase (*ChAT*) (Millipore, dilution 1:50) in BMMs stimulated with M-CSF and RANKL and (*right panel*) control staining with secondary antibody (*AB*) (biotinylated rabbit-antigoat IgG) only. Scale bars = 100 μm. **b**–**g** Effect of nAChR ligands on osteoclastogenesis. Images in the *bottom panel* are representative of corresponding experiments; scale bar = 100 μm; **b** nicotine; **c** epibatidine; **d** PNU282987; **e** nicotine after pretreatment with 100 μM mecamylamine; **f** mecamylamine; **g** alpha-bungarotoxin. *BTX* alphabungarotoxin; *TRAP* tartrate-resistant acidic phosphatase

We first isolated cells from the bone marrow of mice and stimulated them with M-CSF to generate BMMs. We then showed that following stimulation with M-CSF and RANKL these cells stain positively for choline-acetyltransferase, the enzyme responsible for acetylcholine production (Fig. 1a). Moreover, in line with previous findings [12, 17], we could demonstrate that BMMs initially stimulated with M-CSF and subsequently with both M-CSF and RANKL express mRNA of several alpha nAChR subunits, including α subunits, 2–7, 9 and 10 as well the beta subunits 2–3 (Additional file 2: Figure S1), implying a potential role of not only homomeric but also heteromeric nAChRs on osteoclastogenesis.

In order to evaluate nAChRs, we utilized both non-specific agonists (nicotine, epibatidine) as well as a specific agonist (PNU282987) in the in vitro osteoclastogenesis assay. Interestingly, both the non-specific agonist nicotine but also the α7-specific agonist PNU282987 caused an increase in OC numbers at low doses, which was significant in the case of PNU282987.

However, at higher concentrations all agonists, including epibatidine, caused a marked, dose-dependent inhibition of OC formation (Fig. 1b-d).

We next examined whether the inhibition of OCs caused by the agonists is nAChR-mediated. Therefore, we added the non-selective, non-competitive nAChR antagonist mecamylamine to the culture. However, at lower concentrations mecamylamine was unable to prevent the inhibitory effect of nicotine (Fig. 1e). Moreover, when mecamylamine was administered at high doses, we observed a similar inhibition of OCs as seen with the agonists (Fig. 1f). Interestingly, not only the non-selective antagonist mecamylamine, but also the α7 nAChR selective antagonist alpha-bungarotoxin caused a similar, inhibition of OC formation (Fig. 1g). Taken together we could demonstrate that ligands of nAChRs, including both agonists and antagonists, inhibit OCs in vitro.

### Nicotine reversibly blocks RANKL-induced osteoclast formation in vitro

**Fig. 2:**
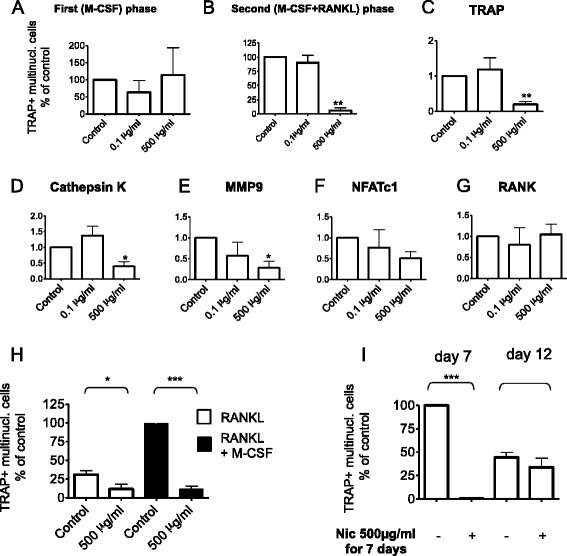
Nicotine reversibly blocks RANKL-induced osteoclast formation in vitro*.* **a** Effect on nicotine on the first (M-CSF-driven) phase of osteoclastogenesis. **b** Effect on nicotine on the second (M-CSF and RANKL-driven) phase of osteoclastogenesis. **c**–**g** Effect of nicotine on expression of RANKL-induced genes: **c** TRAP; **d** cathepsin K; **e** MMP9; **f** NFATc1; **g** RANK. **h** Effect of nicotine on osteoclastogenesis in the presence and absence of M-CSF in the second phase. **i** Osteoclast formation after 7 days of treatment with 50 ng/ml RANKL and 30 ng/ml M-CSF in the presence/absence of nicotine 500 μg/ml; osteoclast formation after 12 days of treatment with 50 ng/ml RANKL and 30 ng/ml M-CSF with presence/absence of nicotine 500 μg/ml in the first 7 days of the assay. *M-CSF* Macrophage colony-stimulating factor, *MMP9* matrix-metalloprotease 9, *NFATc1* nuclear factor of activated T-cells, cytoplasmic, calcineurin-dependent 1, *Nic* nicotine, *RANK* receptor activator of nuclear factor kappa B, *RANKL* receptor activator of nuclear factor kappa B ligand, *TRAP* tartrate-resistant acidic phosphatase

In order to further elucidate this blocking effect, we added the nAChR agonist nicotine either to the first, M-CSF-driven, and/or to the second, predominantly RANKL-driven, phases of osteoclastogenesis. When we administered nicotine parallel only to the first, but not the second, phase we observed no inhibition of OC formation (Fig. 2a). However, when the agonist at a high-dose was added concomitant to the second, M-CSF and RANKL-driven, phase we detected a marked reduction in OC numbers (Fig. 2b). Moreover, this effect was accompanied by reductions in the relative expression of RANKL-dependent genes including TRAP, cathepsin K, and matrix metalloproteinase 9 (Fig. 2c–e); we found, however, no significant change in the expression of receptor activator of nuclear factor kappa B (RANK) and NFATc1 (Fig. 2f and g).

To elaborate the blocking effect of nicotine on the second phase in more detail we performed experiments in which we administered RANKL without M-CSF, whereupon we observed a decrease in OC numbers which was further reduced upon administration of high-dose nicotine together with RANKL (Fig. 2h).

In order to assess the reversibility of the effect of high-dose nicotine on osteoclastogenesis we performed experiments where at the end of our standard in vitro osteoclastogenesis assay (day 7) we cultured the cells further with M-CSF and RANKL for 5 additional days. We could observe that when nicotine was removed from the medium after the end of the standard assay on days 7 and cells were cultured further with M-CSF and RANKL osteoclastogenesis was recovered (Fig. 2i). Hence, effects of nicotinic agonists on OC formation appear to be reversible.

### Nicotine inhibits in vivo osteoclastogenesis

**Fig. 3:**
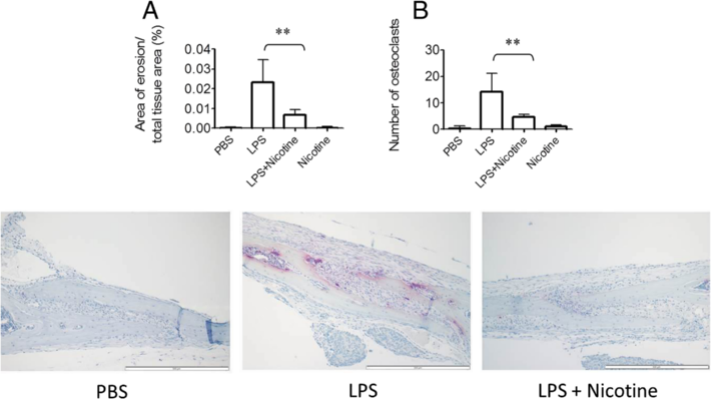
Nicotine inhibits in vivo osteoclastogenesis. Calvarial bone resorption induced by subcutaneous injection of lipopolysaccharide (*LPS*) above the calvaria; LPS, phosphate-buffered saline (*PBS*), nicotine, or nicotine and LPS. Images in the *bottom panel* are representative of the indicated groups; scale bars = 100 μm. **a** Area of erosion/total tissue area; **b** number of osteoclasts

We next evaluated the effect of the nAChR ligand nicotine on in vivo osteoclastogenesis. We induced osteoclastogenesis by injecting LPS subcutaneously above the calvariae of WT mice. In mice treated with LPS we observed bone destruction accompanied by osteoclastogenesis, which was significantly reduced when nicotine was administered concomitantly to LPS via subcutaneous injection (Fig. 3).

### nAChR ligands block differentiation of TRAP-positive mononuclear cells in vitro

**Fig. 4:**
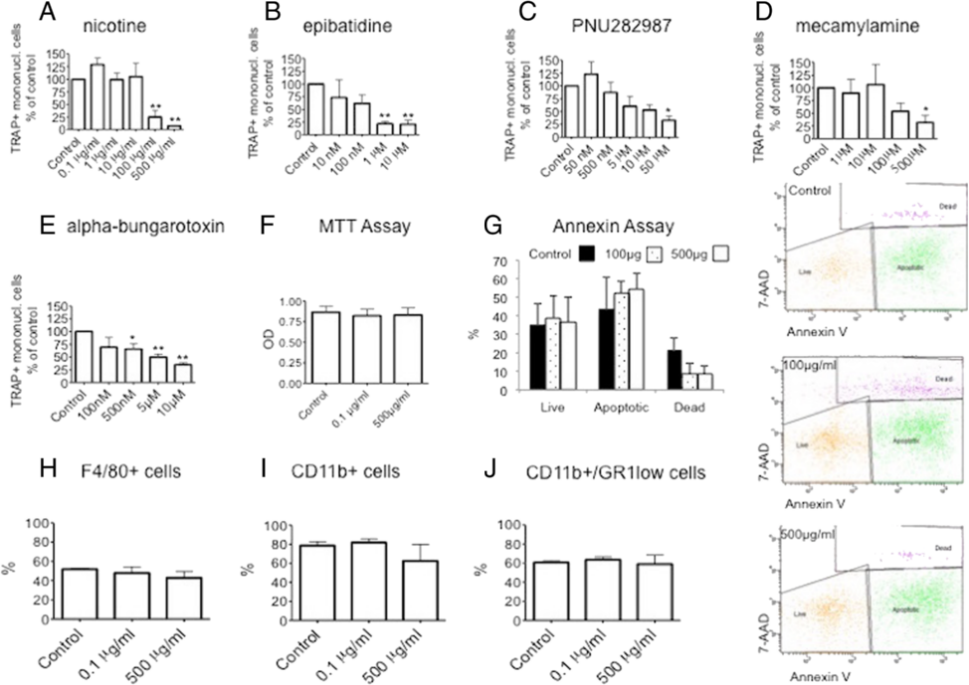
Nicotinic AChR ligands block the differentiation of TRAP-positive mononuclear cells in vitro*.* **a** Nicotine; **b** epibatidine; **c** PNU282987; **d** mecamylamine; **e** alpha-bungarotoxin; **f** effect of nicotine on cytoxicity as assessed by MTT assay; **g** percentage of live (Annexin V low/7-AAD low), apoptotic (Annexin V high/7-AAD low) and dead (Annexin V high/7-AAD high) cells with flow cytometry plots showing representative experiments (*right panels*). **h**–**j** Effect of nicotine on percentage of cells at the end of the in vitro osteoclastogenesis assay staining positive for marker: **g** F4/80; **h** CD11b; **i** GR1 low/CD11b+. *7-AAD* 7-Aminoactinomycin-D, *OD* optical density, *TRAP* tartrate-resistant acidic phosphatase

Having shown that nAChR ligands block OC formation in vitro by interfering with RANKL, we next addressed the question whether nAChR ligands also affected the differentiation of TRAP-positive mononuclear cells, which may fuse to become osteoclasts. Indeed we found that both non-selective (nicotine, epibatidine) and selective nAChR agonists (PNU282987) caused a dose-dependent, significant decrease in the number of TRAP-positive mononuclear cells (Fig. 4a–c), which we could also observe upon treatment with the non-selective nAChR antagonist mecamylamine and selective nAChR antagonist alpha-bungarotoxin (Fig. 4d and e).

Treatment with nicotine even at the highest concentration (500 μg/ml) did not affect cell-surface expression of the classical monocyte and granulocyte markers F4/80, CD11b and GR1 as assessed by flow cytometry (Fig. 4g–i). We assessed cytotoxicity with the MTT assay where we observed no difference between control conditions and nicotine treatment (Fig. 4f). Based on 7AAD-Annexin V staining, we saw no significant difference in the percentage of live, apoptotic and dead cells between control conditions and nicotine treatment (Fig. 4g).

### Osteoclastogenesis is reduced in knockout mice lacking selected nAChR subunits

Having demonstrated marked effects of nAChR ligands on osteoclastogenesis in vitro, we next evaluated which nAChR receptor subunits might be involved in this inhibition. Taking into account that all heteromeric nAChRs include a beta subunit, we evaluated mice lacking the beta2 subunit (β2–/–) because this subunit was expressed in BMMs stimulated with RANKL and M-CSF and because it is a subunit essential for one of the major nAChRs, the central nervous system-type (α4β2) nAChR. We also evaluated animals lacking the beta4 subunit (β4–/–), which was not expressed, but which is an essential component of the ganglion-type (α3β4) nAChR.

**Fig. 5:**
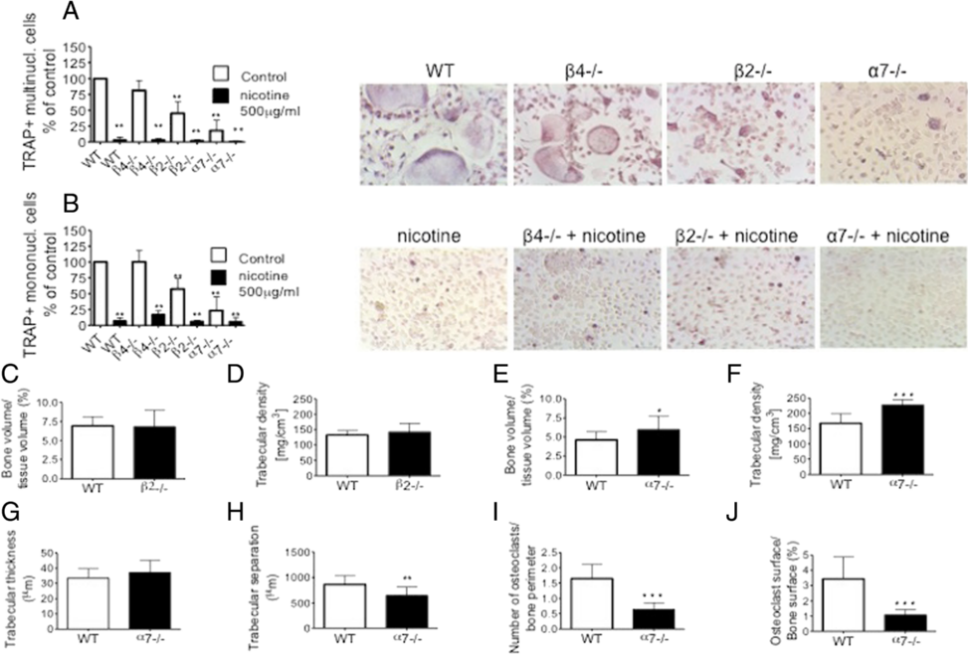
Osteoclastogenesis is partly inhibited in nAChR subunit –/– mice. **a**, **b** Osteoclastogenesis in WT, α7–/–, β2–/– and β4–/– mice with or without 500 μg/ml nicotine treatment. Images in the *right panel* are representative of the corresponding experiments; scale bars = 100 μm. **c**, **e** Bone volume/tissue volume; **d**, **f** trabecular density; **g** trabecular thickness; **h** trabecular separation; **i** number of osteoclasts/bone perimeter; **j** osteoclast surface/bone surface. *TRAP* tartrate-resistant acidic phosphatase, *WT* wild-type

While we found no differences in the number of OCs or TRAP-positive mononuclear cells in osteoclastogenesis cultures from β4–/– mice as compared to those generated from WT littermates, the numbers of both types of cells were significantly reduced in cultures generated from β2–/– mice (Fig. 5a and b). However, this inhibitory effect observed on osteoclastogenesis in vitro had no correlate in vivo*,* since bone volume or trabecular density in β2–/– animals were not different from WT littermates (Fig. 5c and d).

Among homomeric nAChRs, the α7 subunit has particular significance in non-neuronal cells; we therefore evaluated osteoclastogenesis in mice lacking this subunit (α7–/– mice). In vitro we found decreased numbers of OCs and TRAP-positive mononuclear cells in osteoclastogenesis cultures generated from α7–/– mice compared to those generated from WT littermates (Fig. 5a and b).

We next analyzed the tibial bones of male and female α7–/– animals. We found no significant difference in BMD or in histomorphometrical analysis in female α7–/– animals. However we found increased trabecular density (+26 %) as assessed by microcomputed tomography, accompanied by increased bone volume/tissue volume (+23 %) and reduced trabecular separation by histomorphometric analysis (Fig. 5e–h) in male α7–/– animals. This finding was further supported by a reduced number of osteoclasts/bone perimeter and reduced osteoclast surface/bone surface, in line with a high bone mass phenotype in vivo (Fig. 5i and j). We found no difference between the number of osteoblasts/bone perimeter and osteoblast surface/bone surface between any α7–/–, β2–/– mice and WT littermates (Additional file 3: Figure S2).

To evaluate whether the ligands might be acting on nAChRs in general rather than on specific subunits, we treated osteoclastogenesis cultures generated from the knockout mice with nicotine and observed similar inhibition of osteoclastogenesis as seen in cultures generated from WT littermates (Fig. 5a and b). Osteoclastogenesis cultures from α7–/–/β2–/– double knockout animals did not differ from cultures prepared from α7–/– mice (data not shown).

### nAChR ligands inhibit RANKL-induced Ca^2+^ oscillation and activation of NFATc1

Nicotinic AChRs are ion channel receptors which permit the movement of cations, including Ca^2+^ [5]. Oscillations of the intracellular Ca^2+^ level in turn seem to play an essential role in mediating the effect of RANKL, as osteoclastogenesis is impaired in virtually all cases when Ca^2+^ oscillation is not induced [32–34]. We therefore evaluated the potential effects of nAChR ligands on RANKL-induced Ca^2+^ oscillation.

**Fig. 6:**
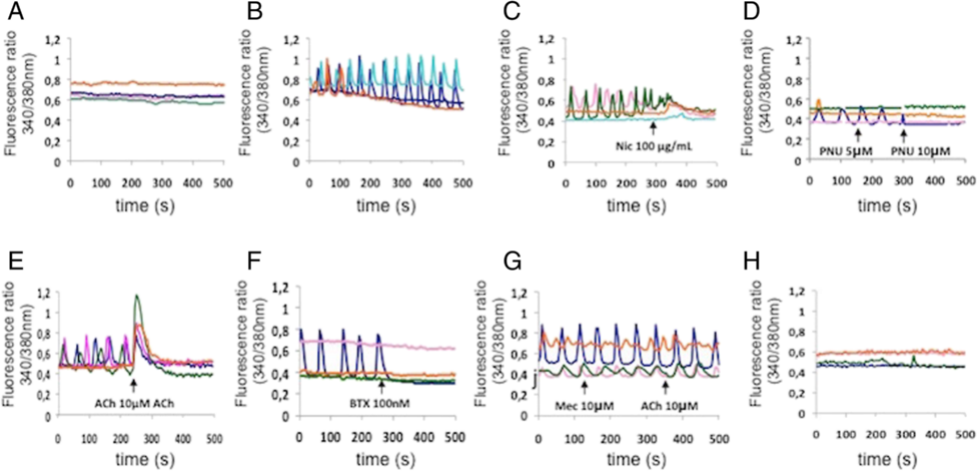
Nicotinic AChR ligands inhibit RANKL-induced Ca^2+^ oscillation. **a**, **b** Intracellular Ca^2+^ level in BMMs, **a** cultured in the presence of M-CSF for 72 hours and **b** cultured in the presence of M-CSF and RANKL for 72 hours. **c**–**g** Effect of acute administration of nAChR ligands on intracellular Ca^2+^ level in BMMs cultured in the presence of M-CSF and RANKL for 72 hours. **c** Nicotine (*Nic*; 100 μg/ml); **d** PNU282987 (*PNU*; 5–10 μM); **e** acetylcholine (*Ach*; 10 μM); **f** alpha-bungarotoxin (*BTX*: 100 nM); **g** mecamylamine (*Mec*; 10 μM) and ACh (10 μM). **h** Intracellular Ca^2+^ levels in BMMs cultured in the presence of M-CSF, RANKL and nicotine (100 μg/mL) for 72 hours

First we found that BMMs cultured in the presence of M-CSF and RANKL for 3 days showed characteristic Ca^2+^ oscillation after 72 hours of treatment with RANKL, while the cells cultured in the presence of M-CSF alone showed no alterations in intracellular calcium levels (Fig. 6a and b). When we induced Ca^2+^ oscillation by pre-treating BMMs with RANKL and thereafter administered the agonists nicotine and PNU-282987, we observed an immediate inhibition of RANKL-induced Ca^2+^ oscillation (Fig. 6c and d). Of note, we saw the same effect when we administered the physiological ligand of nAChR, acetylcholine (ACh) (Fig. 6e). To evaluate the nature of this inhibition, we added the antagonist alpha-bungarotoxin, which caused a similar effect (Fig. 6f). We next added mecamylamine shortly before treating oscillating BMMs with ACh and found that ACh failed to inhibit Ca^2+^ oscillation under these conditions (Fig. 6g). BMMs subjected to prolonged exposure (3 days) with high-dose nicotine (100 μg/ml) and RANKL, administered together, failed to induce the characteristic Ca^2+^ oscillations (Fig. 6h).

We next asked whether this blockade also affected downstream elements of the RANK signaling pathway, such as the transcription factors NFATc1 and c-fos [35, 36]. Therefore, we isolated protein from osteoclastogenesis cultures at various time points throughout the assay and performed Western blot experiments. Indeed, nicotine inhibited the induction of c-fos after 24-hour stimulation with RANKL, and of NFATc1 after 24-hour, 48-hour or 7 days of stimulation with RANKL (Additional file 4: Figure S3).

## Discussion

The RANK receptor and its ligand, RANKL, play key roles in osteoclastogenesis both within the framework of physiological bone turnover and in various diseases characterized by inflammatory bone loss [16]. The central role of RANKL in osteoclast formation and bone degradation is further underlined by successful therapeutic approaches targeting this pathway. Denosumab, a RANKL-specific monoclonal antibody, is not only an effective treatment for postmenopausal osteoporosis, but also reduces local bone loss in patients with rheumatoid arthritis [37, 38]. The finding that nAChR activation can interfere with RANK signaling is supported by the following findings: 1) when nicotine was added only to the first (solely M-CSF-driven) phase of osteoclast differentiation, it had no effect on OC generation; 2) when nicotine was present during the second phase in parallel with MCSF and RANKL, it caused an inhibitory effect; 3) even when M-CSF was absent from the second phase, nicotine was able to reduce osteoclastogenesis; and 4) the relative expression of RANKL-dependent gene, including TRAP, cathepsin K, matrix metalloproteinase 9 and NFATc1, was decreased in OCs treated with nicotine.

Herein we demonstrate that nAChRs are crucially involved in RANKL-induced osteoclastogenesis by inhibiting Ca^2+^ oscillations, which in turn blocks RANKL-induced activation of c-fos and NFATc1. In contrast to our findings, it has been recently suggested that nAChR agonists have no effect on osteoclastogenesis in vitro [12]; however, the ligands were used at lower concentrations than we utilized in our study. Tanaka et al. administered nicotine at doses comparable to those used here, and observed a reduction in the number of large OCs and the resorptive index, but an increase in the number of small OCs suggesting a reduction in fusion [17]. We were also able to observe an increase in the number of osteoclasts upon administration of low-dose nicotine and PNU282987. Thus nicotinic agonists at low concentration may favor the generation of osteoclasts, whereas high concentrations inhibit osteoclastogenesis. Interestingly it has been suggested that different nAChRs may also convey opposing effects as evidenced by recent studies, which described increased in vitro osteoclastogenesis as well as a significant, though modest, decrease in the systemic bone mass of α2–/– mice [12] and increased bending stiffness and cortical thickness in female α7–/– mice, while male counterparts showed no difference as compared to WT [20]. However, male α7 β2–/– exhibited a significant increase in BMD when compared to WT mice [39]. Interestingly, we found an, albeit modest, increased bone mass in male α7–/– mice, which we could not demonstrate in female α7–/– mice. Gender disparity with regard to bone phenotype is not uncommon and has been described among others in CB1–/– or GPR55–/– mice [40, 41]. In both of these cases males exhibited an osteopetrotic phenotype which females did not, and which was not otherwise explainable. In line with previous studies, which showed no change in gene expression of osteoblast markers, we found no change in the osteoblast phenotype in α7–/– mice [19, 20]. Taken together it appears that high levels of nAChR stimulation may have an overall negative effect on osteoclastogenesis but actions mediated via the α7 receptor may indeed be different and positive in nature.

Our observation that nAChR antagonists also inhibit osteoclastogenesis, coupled with our results showing reduced osteoclastogenesis in α7–/– mice, can be interpreted in the context of desensitization, a key property of nAChRs. Depending on the receptor subtype and the type of agonist, as well as the duration of exposure to nAChRs, agonists have been shown to alter the affinity state of the receptor inducing desensitization, leading to a functional blockade of the receptor [42, 43]. In particular, nicotine and certain experimental α7-selective partial agonists are known to produce a transient activation of α7 receptors followed by a period of prolonged residual inhibition or desensitization [44]. While we derived the dosage of nicotine from published studies [17, 18], due to the fact that we used high doses we cannot exclude the possibility that the inhibition seen with the high-dose nAChR ligands is due to either effects on mAChRs, non-receptor mediated effects (such as the blocking of additional ion channels), or to other factors involved in the regulation of osteoclastogenesis.

Oscillating levels of intracellular calcium appear to be critical for osteoclastogenesis primarily by inducing the dephosphorylation and shuttling of NFATc1 into the nucleus, where it plays a decisive role in the regulation of osteoclastogenesis [32–34]. After observing the marked acute effect of both nAChR agonists and antagonists on Ca^2+^ oscillation, we could show that, indeed, the nAChR agonist nicotine inhibited RANKL-induced expression and production of NFATc1 and also had a similar effect on c-fos, an early inducer of NFATc1 [45].

The fact that cholinergic signaling has an effect on osteoclastogenesis is also supported by studies which have demonstrated that ACh is produced by cells of the immunoinflammatory sytem, including lymphocytes [46], and the fact that bone marrow also possesses its own sensory and autonomic innervation including cholinergic neurons [12, 14, 15]. This suggests that, under certain circumstances, in addition to ACh released from BMMs, differentiating OCs may also be exposed to ACh released from neurons, which could then have various effects on their nAChRs.

## Conclusions

Our results suggest that cholinergic agonists inhibit RANKL-induced osteclastogenesis by interfering with intracellular calcium levels and consequently with the NFATc1 signaling pathway. Of note, this complete blockade of osteoclastogenesis in vitro was neither due to toxic effects nor associated with apoptosis, and was shown to be reversible. However, while high levels of nAChR activity appear to inhibit RANKL-induced osteoclastogenesis, actions mediated by certain nAChRs, in particular the α7 homomeric receptor, may favor it. Further studies are needed to determine the gender-specific effect of α7 and other nAChRs and mAChRs on bone homeostasis.

## Additional files

Additional file 1: Table S1. Primer sequences of genes investigated with quantitative real time PCR. (JPEG 27 kb) Additional file 2: Figure S1. Semi-quantitative real-time PCR analysis of nicotinic acetylcholine receptor subunits in murine osteoclasts and nucleus interpeduncularis. Murine bone marrow-derived osteoclasts (O); nucleus interpeduncularis (I); – represents negative control; * represent lanes with molecular weight ladder; nicotinic acetylcholine receptor subunits are designated by respective Greek letters and Arabic numerals. (JPEG 89 kb) Additional file 3: Figure S2. Osteoblast phenotype in nAChR knockout animals and wildtype littermates. A: number of osteoblasts/bone perimeter; B: osteoblast surface/bone surface. (JPEG 47 kb) Additional file 4: Figure S3. Nicotine inhibits induction of NFATc1 and c-fos. A, B: Western blot bands and quantification. Protein extracts obtained from mouse bone marrow-derived macrophages cultured in the presence of 30 ng/ml M-CSF (C), 30 ng/ml M-CSF and 50 ng/ml RANKL (R), 30 ng/ml M-CSF and 500 μg/ml nicotine (N), 30 ng/ml M-CSF and 50 ng/ml RANKL and 500 μg/ml nicotine (R + N) for the indicated timepoints; A: NFATc1 and actin; B: c-fos and actin. NFATc1:actin and c-fos:actin ratios are shown in bar graphs in the bottom panel. (JPEG 88 kb)

## Abbreviations

7-AAD: 7-Aminoactinomycin-D
ACh: Acetylcholine
BMD: Bone mineral density
BMM: Bone marrow-derived macrophage
cDNA: Complementary DNA
EDTA: Ethylenediaminetetraacetic acidl
FITC: Fluorescein-isothiocyanate
LPS: Lipopolysaccharide
mAChR: Muscarinic acetylcholine receptor
M-CSF: Macrophage colony-stimulating factor
nAChR: Nicotinic acetylcholine receptor
NFATc1: Nuclear factor of activated T-cells, cytoplasmic, calcineurin-dependent 1
OC: Osteoclast
PBS: Phosphate-buffered saline
pQCT: Peripheral quantitative computed tomography
RANK: Receptor activator of nuclear factor kappa B
RANKL: Receptor activator of nuclear factor kappa B ligand
RT-PCR: Real-time polymerase chain reaction
TRAP: Tartrate-resistant acidic phosphatase
WT: Wild-type
