# Supplementary figures and images for: Nicotinic acetylcholine receptors modulate osteoclastogenesis
Source: Arthritis Res Ther. 2016 Mar 12;18:63. doi: 10.1186/s13075-016-0961-x (PMC4789270; doi:10.1186/s13075-016-0961-x)

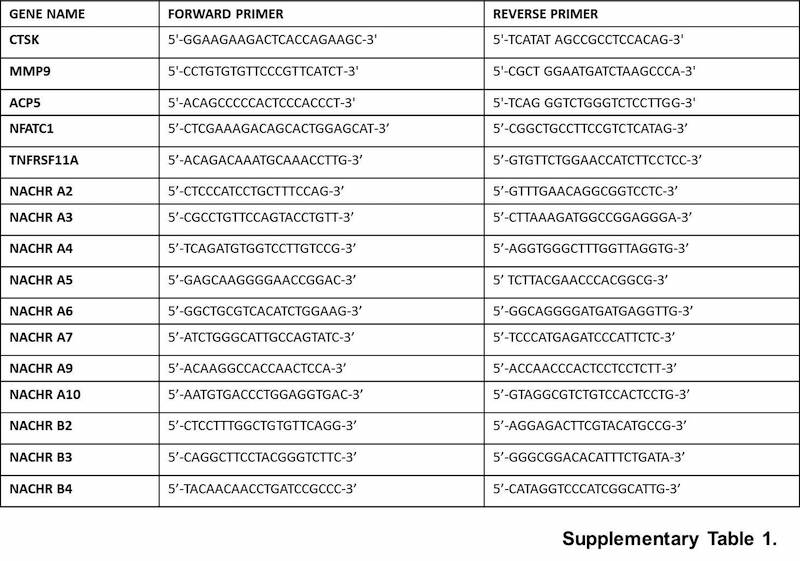

Supplement: Additional file 1: Table S1. — Primer sequences of genes investigated with quantitative real time PCR. (JPEG 27 kb) [file 13075_2016_961_MOESM1_ESM.jpeg]

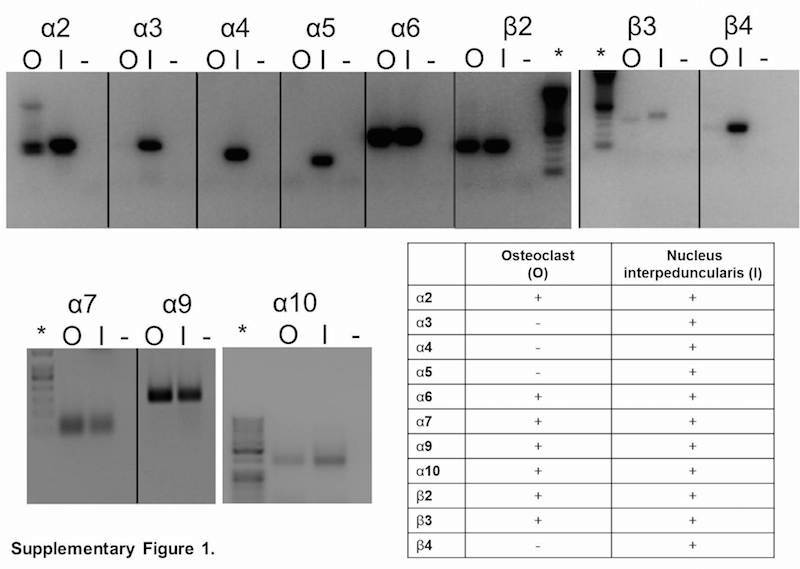

Supplement: Additional file 2: Figure S1. — Semi-quantitative real-time PCR analysis of nicotinic acetylcholine receptor subunits in murine osteoclasts and nucleus interpeduncularis. Murine bone marrow-derived osteoclasts (O); nucleus interpeduncularis (I); – represents negative control; * represent lanes with molecular weight ladder; nicotinic acetylcholine receptor subunits are designated by respective Greek letters and Arabic numerals. (JPEG 89 kb) [file 13075_2016_961_MOESM2_ESM.jpeg]

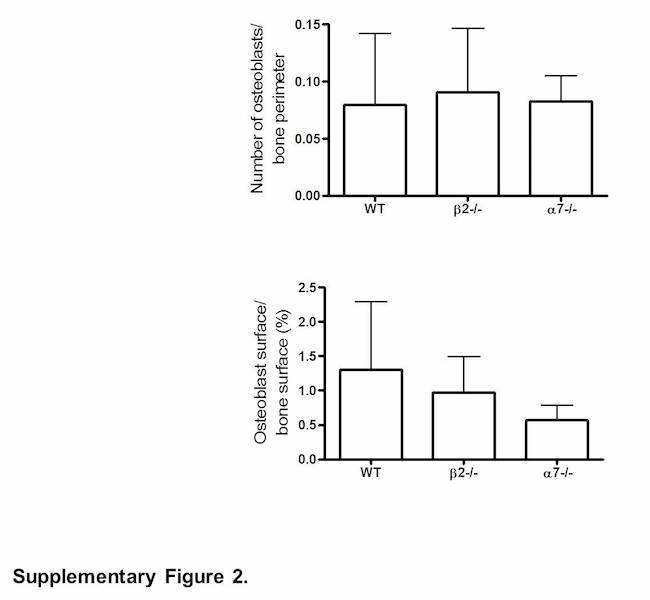

Supplement: Additional file 3: Figure S2. — Osteoblast phenotype in nAChR knockout animals and wildtype littermates. A: number of osteoblasts/bone perimeter; B: osteoblast surface/bone surface. (JPEG 47 kb) [file 13075_2016_961_MOESM3_ESM.jpeg]

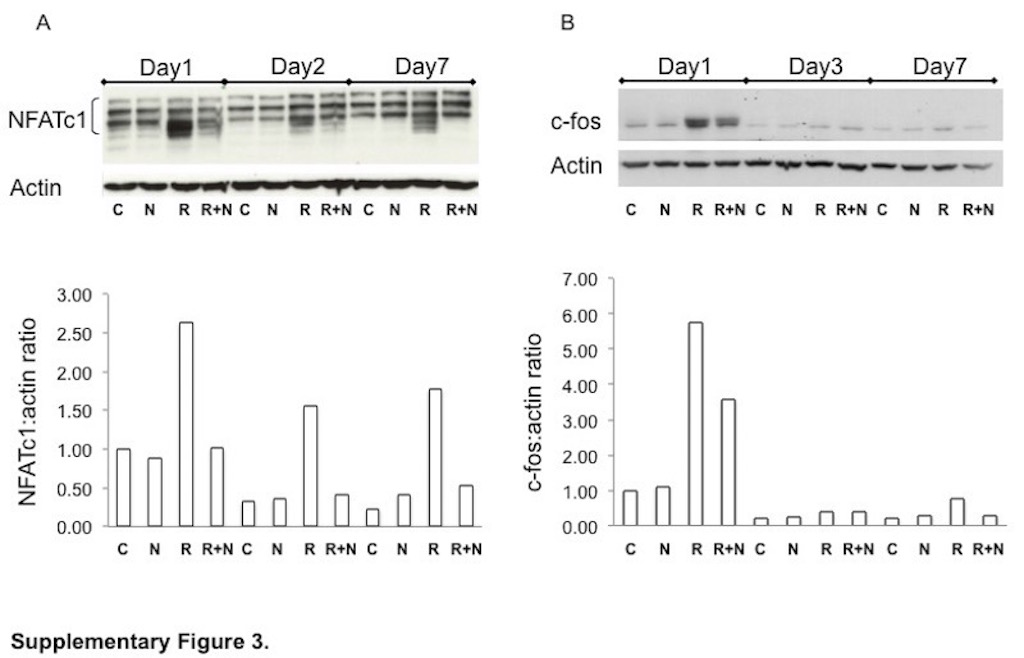

Supplement: Additional file 4: Figure S3. — Nicotine inhibits induction of NFATc1 and c-fos. A, B: Western blot bands and quantification. Protein extracts obtained from mouse bone marrow-derived macrophages cultured in the presence of 30 ng/ml M-CSF (C), 30 ng/ml M-CSF and 50 ng/ml RANKL (R), 30 ng/ml M-CSF and 500 μg/ml nicotine (N), 30 ng/ml M-CSF and 50 ng/ml RANKL and 500 μg/ml nicotine (R + N) for the indicated timepoints; A: NFATc1 and actin; B: c-fos and actin. NFATc1:actin and c-fos:actin ratios are shown in bar graphs in the bottom panel. (JPEG 88 kb) [file 13075_2016_961_MOESM4_ESM.jpeg]
